# Ultra-Deep Pyrosequencing of Partial Surface Protein Genes from Infectious Salmon Anaemia Virus (ISAV) Suggest Novel Mechanisms Involved in Transition to Virulence

**DOI:** 10.1371/journal.pone.0081571

**Published:** 2013-11-26

**Authors:** Turhan Markussen, Hilde Sindre, Christine Monceyron Jonassen, Torstein Tengs, Anja B. Kristoffersen, Jon Ramsell, Sanela Numanovic, Monika J. Hjortaas, Debes H. Christiansen, Ole Bendik Dale, Knut Falk

**Affiliations:** 1 Department of Laboratory Services, Norwegian Veterinary Institute, Oslo, Norway; 2 Department of Health Surveillance, Norwegian Veterinary Institute, Oslo, Norway; 3 National Reference Laboratory for Fish Diseases, Food and Veterinary Authority, Torshavn, Faroe Islands; Saint Louis University, United States of America

## Abstract

Uncultivable HPR0 strains of infectious salmon anaemia viruses (ISAVs) infecting gills are non-virulent putative precursors of virulent ISAVs (vISAVs) causing systemic disease in farmed Atlantic salmon (*Salmo salar*). The transition to virulence involves two molecular events, a deletion in the highly polymorphic region (HPR) of the hemagglutinin-esterase (HE) gene and a Q_266_→L_266_ substitution or insertion next to the putative cleavage site (R_267_) in the fusion protein (F). We have performed ultra-deep pyrosequencing (UDPS) of these gene regions from healthy fish positive for HPR0 virus carrying full-length HPR sampled in a screening program, and a vISAV strain from an ISA outbreak at the same farming site three weeks later, and compared the mutant spectra. As the UDPS data shows the presence of both HE genotypes at both sampling times, and the outbreak strain was unlikely to be directly related to the HPR0 strain, this is the first report of a double infection with HPR0s and vISAVs. For F amplicon reads, mutation frequencies generating L_266_ codons in screening samples and Q_266_ codons in outbreak samples were not higher than at any random site. We suggest quasispecies heterogeneity as well as RNA structural properties are linked to transition to virulence. More specifically, a mechanism where selected single point mutations in the full-length HPR alter the RNA structure facilitating single- or sequential deletions in this region is proposed. The data provides stronger support for the deletion hypothesis, as opposed to recombination, as the responsible mechanism for generating the sequence deletions in HE.

## Introduction

Infectious salmon anaemia virus (ISAV) is an orthomyxovirus that has caused systemic infection and disease in farmed Atlantic salmon (*Salmo salar*) in Norway, Canada, Scotland, Shetland Islands, the Faroe Islands, USA and Chile [1-8]. The virus’ two major surface glycoproteins are the hemagglutinin-esterase (HE), responsible for receptor binding and release (RDE), and the fusion protein (F), responsible for virus uptake through the fusion of viral and cellular membranes [9-12]. The HEs from different virulent ISAV strains (vISAVs) often vary in length as determined by the size of their highly polymorphic region (HPR) located in the stalk immediately upstream of the transmembrane domain of the protein. The shortening of the stalk is thought to arise from differential deletions from a full-length precursor HE (HPR0) [13]. Recombination through template switching during replication has been proposed as an alternative mechanism for generating the deletions [14]. The deletions in the HE stalk region could be analogues to the varying lengths in the influenza A virus neuraminidase stalk, which has been associated with host switching [15-18]. A shortening of the HE stalk could also affect the functional balance between the HE receptor-binding and -destroying activities similar to that found between the hemagglutinin and neuraminidase of influenza A viruses through a number of studies [19-23]. 

ISAV HPR0 genotypes containing full-length HEs are non-virulent, non-cultivable and are primarily found in gills [24-26]. They have been detected in apparently healthy wild and farmed Atlantic salmon in most regions with Atlantic salmon farming [24-28]. Frequent findings of HPR0s in fish farms in Norway and the Faroe Islands, the former with few disease outbreaks and the latter with no ISA outbreaks since 2005, suggests the transition to virulence is an infrequent occurrence [27,29]. In addition to a full-length HE, all HPR0s also contain a glutamine in position 266 (Q_266_) next to the one of the two putative cleavage sites in the F protein (R_267_) [30]. In contrast, all vISAVs carry a leucine in this position (L_266_), except for a few strains which have small sequence insertions close to this site [30-32]. F gene insertions through template switching, as well as reassortment of gene segments, have both been linked to virulence [30,31]. For the F protein, the single amino acid substitution or insertion close to R_267_ may be analogous to that in highly pathogenic avian influenza A virus subtypes H5 and H7, where pathogenicity is acquired through the mutational change of cleavage specificity resulting in altered tissue tropism [33,34]. These differences between HPR0s and vISAVs may represent viral adaptation leading to disease in densely populated industrial farming operations.

RNA viruses are known to exist in the host as a swarm of closely related mutant genomes known as quasispecies [35-37]. The molecular basis for this genomic heterogeneity is that replication of RNA viruses is highly error prone since viral RNA-dependent RNA polymerases do not possess proofreading-repair activity [38-40]. Such a mutant spectrum within an individual host, combined with the ability to recombine and reassort their gene segments, can enable these viruses to rapidly adapt in response to selective pressures [36,37]. This genetic diversity can also be viewed as a measure of the fitness of the virus and be linked directly to pathogenesis [41-43]. Ultra-deep pyrosequencing (UDPS) allows for the simultaneous analysis of thousands of clonally amplified PCR amplicons, enabling the detection of the within-host minority variants [44-49]. The technology is now well established in many fields within virology, including viral evolution and pathogenesis, detection of antiviral resistance markers and diagnostics. For ISAV, aside from a recent publication describing the presence of quasispecies in the non-coding regions of the viral genome, based on traditional cloning and Sanger sequencing [50], the within-host genomic heterogeneity for this virus has not been investigated.

An ISA epidemic from 2007 to 2010 in Astafjord of Norway was monitored by both disease diagnostics and screening of apparently healthy fish. Sequence-based analysis of partial HE- and F genes combined with epidemiological information concluded that a single virulent ISAV strain had transmitted horizontally between proximal fish farms and caused all 17 ISA outbreaks in the region from 2007 to 2009 (i.e. Astafjord strain) [28,51]. Here we present results from UDPS of amplicons from gene regions spanning the HPR in HE and the putative R_267_ cleavage site in the F protein. From a site in Astafjord 2010, mutant spectra originating from gill tissue samples found screening positive for a non-virulent HPR0 are compared with that of a vISAV from an ISA outbreak at the same farming site three weeks later. We report, for the first time, a double infection involving a HPR0 and a vISAV. F reads containing L_266_- and Q_266_ codons in screening and outbreak samples, respectively, were present at mutational frequencies comparable to that found at any random site in the amplicons. Similarly, HE reads from screening samples containing deletions within the full-length HPR, differing from the HPR deletion pattern of the outbreak strain (delHPR, present at very low levels) were not observed at all. The results from UDPS data analyses, together with RNA structure predictions, provide stronger support for the HE deletion hypothesis over recombination in creating the sequence deletions in vISAVs, and we propose a mechanism in which selected single point mutations in the full-length HPR alter the RNA structure in such a way that it facilitates single- or sequential deletions in this region of the HE gene.

## Materials and Methods

### Field Samples

Four HPR0-positive gill samples from healthy Atlantic salmon taken from a commercial farming site (hereafter named Site 10) in Astafjord (Norway) during screening in May 2010 (screening samples 1-4), and four gill samples taken three weeks later during the early phases of an ISA outbreak at the same site (outbreak samples 1-4), were chosen for UDPS. Sampling from both screening and disease outbreak, which were from different fish and different locations at the site, were performed on order by the Norwegian Food Safety Authority. Approval from Institutional Animal care and Use Committee (IACUC) or ethics was not required. No experiments that involved fish were performed. The screening samples were among 8 of 23 gill samples ISAV positive by real-time RT-PCR (Ct’s 28-38, not shown). Traditional (Sanger) sequencing of HE- and F genes, covering the HPR and the putative encoded R_267_ cleavage site, respectively, showed all to be of one non-virulent HPR0 genotype [29]. The ISA outbreak fish had pathology consistent with ISA, and the systemic infection was verified by real-time RT-PCR and Sanger sequencing of the HE gene from kidney samples of ten fish tested (Ct’s 14-18, not shown). Real-time RT-PCR to determine viral loads in gill samples selected for UDPS was performed as previously described [28].

### Preparation of PCR-generated Amplicons

Gill tissue was homogenized and total RNA extracted using RNeasy Mini Kit (Qiagen, Hilden, Germany). cDNA synthesis was performed separately for each sample using random hexamers together with Superscript III reverse transcriptase, following the protocol recommended by the manufacturer (Invitrogen, Carlsbad, USA). Following first-strand synthesis, cDNA samples were used as templates in two separate PCR reactions to generate gene segment 5 (F gene) and segment 6 (HE gene) amplicons. Primer sequences are shown in Table **S1**. PCR was performed using high fidelity AccuPrime *Pfx* DNA polymerase (Invitrogen, Carlsbad, USA) with 2 µl cDNA in each reaction. PCR cycling conditions were 95°C/2 min, followed by 40 cycles of 95°C/45 sec, 55°C/45 sec and 68°C/2 min and a final extension step at 72°C/7 min. After PCR, a small volume from each reaction was run on 1.5 % agarose gel electrophoresis and the products visualized by ethidium bromide staining. In some cases the 2100 Bioanalyzer (Agilent Technologies) was also used to evaluate the quality of PCR products. For most samples, single PCRs produced sufficient amount of product, except for screening samples 2, 3 and 4 where mixing products from several parallel PCRs was necessary in order obtain the recommended amount of each product for pyrosequencing. The same cDNA sample was used in each PCR parallel. Samples from PCR were treated with ExoSAP-IT to remove unincorporated dNTPs and primers, following manufacturer instructions (Affymetrix). Purification was performed using the QIAquick PCR Purification kit (Qiagen, Hilden, Germany). Quantification was performed using Nanodrop 2000 (Thermo Scientific). In addition to pyrosequencing each amplicon in both directions, the eight purified HE- and F gene products were adjusted to equimolar amounts for multiplexing. Finally, two single samples (screening sample 1 and outbreak sample 1) and two pooled samples (screening samples 2-4 and outbreak samples 2-4) were prepared and distributed over four regions on a Genome Sequencer FLX (GS-FLX) Titanium platform (Roche) at the Norwegian Sequencing Centre, University of Oslo.

### Data Analysis

The CLC Genomics Workbench program (CLC bio) was used for assembly of sequences. Prior to in-depth analysis of the pyrosequencing data, the reads were split only accepting reads containing 0 errors in the multiplex identifiers (MIDs). Sequences were then de-multiplexed, and adaptor sequences, keys, MIDs and primer ends trimmed away. This would, when compared to the Sanger sequence lengths (reference), correspond to amplicon sizes of 250 nucleotides (nts) (HPRO HE), 187 nts (delHPR HE) and 194 nts (F). The comparative aspect between the screening and outbreak samples was the main focus of the present work. In order to avoid low-frequency mutational artifacts from the plasmid RT-step and risk of cross contamination during UDPS setup, which could mask true quasispecies present at low frequencies, *in vitro* transcribed plasmid cloned RT-PCR-amplified templates were not included as controls. The “RNA environments” as represented by short identical RNAs (from controls) and total RNA (from samples) could also introduce differential mutational artifacts following RT-PCR amplification. Therefore, a site by site manual comparison of observed frequency of nucleotides differing from the Sanger sequences was chosen as an alternative for all reads from screening and outbreak samples. Based on the results from these analyses a mutational cutoff value of 0.05% was chosen in order to identify mutations occurring only at particular high frequencies. Potential sequencing errors related to homopolymers was addressed by excluding variable sites present as stretches of four or more identical nucleotides. In addition, mutation frequencies above cutoff, as well as reads containing sequence deletions and/or insertions (see below), would have to be present in both forward and reverse directions in order to be considered. When an estimation of the number of reads containing a particular deletion was to be determined, the minimum length fraction setting was always set to 1.0 while the minimum similarity fraction was set to 0.99. This would allow no flexibility with regards to length of the reads, but would allow for 1 to 3 mismatches, thus providing a necessary flexibility with regards to single nucleotide differences in the reads when determining the number of reads that contained the particular deletion type. CLC Main Workbench (CLC bio) and Align X (Vector NTI Advance™ 11 Package, Invitrogen) were used as additional tools for comparisons between individual sequence reads. RNA secondary structure predictions (minimal free-energy models) of viral (+)/(-)RNAs were performed using the Mfold program (version 2.3) [52]. Default parameters were used in the predictions except for temperature, which was set to 15°C, considered the optimal temperature for ISAV replication [53].

### Real-Time PCR on Amplicons

As there was a theoretical possibility that the detection of delHPR reads in screening samples and full-length HPR reads in outbreak samples could have originated from cross-contamination during UDPS setup, TaqMAN real-time PCR was performed on all HE amplicon samples (except screening sample 2, no more sample). The assays were designed specifically to distinguish between full-length HPRs and delHPRs by placing the two reverse primers in this region. First, 1 µl of the amplicon samples used for UDPS was run on a 2% agarose gel for separation of the two size variants. Bands, both visible and estimated, corresponding to full-length- or delHPR, were excised and purified using QIAquick Gel Extraction Kit following manufacturers protocol (Qiagen, Hilden, Germany). Real-time PCR was performed using 5 µl of sample and TaqMAN Universal PCR Master Mix (Invitrogen, Carlsbad, USA). Primer- and probes used are shown in Table **S1**. Real-time PCR was run on a Stratagene Mx3005P (Agilent Technologies) with cycling conditions 95°C/10 min followed by 40 cycles of 95°C/15 sec, 55°C or 60°C/1 min and 72°C/45 sec. Real-time PCR products were run on 2 % agarose gel, bands excised and purified as above, and sequenced using primer s6 HPR_F (Table **S1**) on a ABI 3130 Genetic Analyser (Invitrogen).

### Statistical Approach

To evaluate whether events like mutations or deletions appear more frequently in one region compared to another, a chi-squared test was developed. Here, the actual number of events observed in each region, and the possible numbers of sites represented by the amplicon sizes of each of the regions, were compared. The null hypothesis tested each time was whether the frequencies of events were similar in the two amplicons.

### Accession Numbers

The Sanger nucleotide sequences obtained and used in this study have GenBank accession numbers KC907278-KC907293. The UDPS dataset has been deposited into BioProject database (NCBI) with the accession number PRJNA221196.

## Results

This paper describes the results obtained from UDPS of the variable regions in ISAV HE- and F genes from non-virulent HPR0-positive fish collected from screening, and a virulent strain sampled from an ISA outbreak at the same farming site three weeks later. From UDPS, mutant spectra were compared and sequence data linked to predicted RNA structural properties of the two genes. Table **1** shows the total number of reads (25695 to 88172) obtained from bidirectional UDPS of HE- and F amplicons following trimming and de-multiplexing. On average, the number of reads identical to Sanger sequence was 90% for both HE and F. In the remaining 10%, mutations, deletions and insertions were observed.

**Table 1:** Raw data from UDPS of partial ISAV HE- and F gene amplicons.

ISAV Sample(s)	Sequence direction	No. of trimmed reads	Percent of reads identical to Sanger sequence
SCN1^a^	HE gene forward	50552	82.4
	HE gene reverse	69684	91.4
	F gene forward	44498	91.9
	F gene reverse	49118	89.2
SCN pool	HE gene forward	25695	78.3
(SCN2, SCN3, SCN4)	HE gene reverse	68481	92.2
	F gene forward	31230	92.1
	F gene reverse	35799	89.5
OBK1^b^	HE gene forward	52038	92.4
	HE gene reverse	88172	93.0
	F gene forward	31546	91.4
	F gene reverse	46835	89.7
OBK pool	HE gene forward	40458	92.1
(OBK2, OBK3, OBK4)	HE gene reverse	51772	92.8
	F gene forward	45909	91.9
	F gene reverse	44498	91.9

^a^ SCN=screening sample

^b^ OBK=outbreak sample

### HE Gene Amplicons

#### The majority of high frequency mutations in HE reads were found within the HPR0 full-length HPR

**Figure 1:**
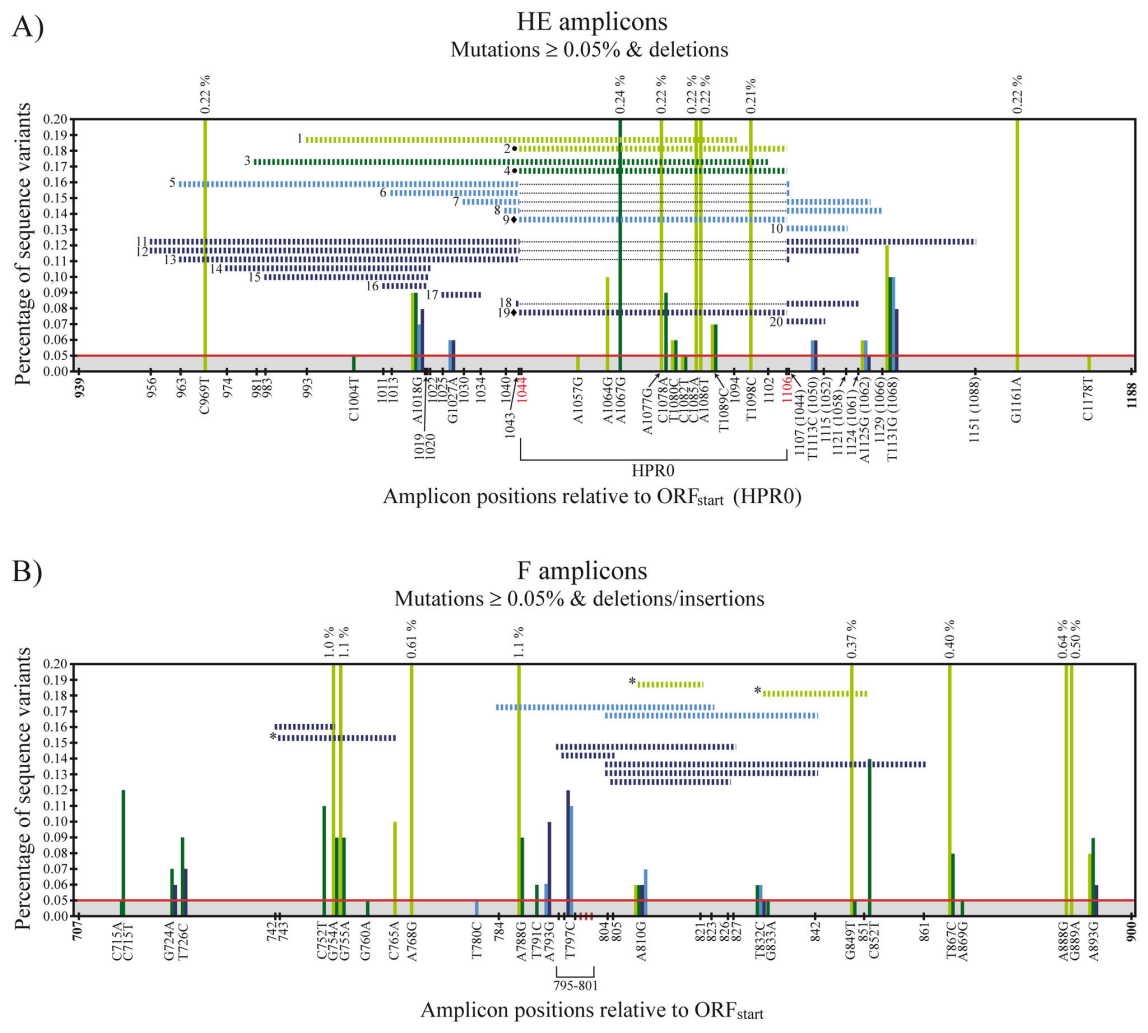
Position, percentage and type of single nucleotide variants (vertical lines), and deletion-insertions and deletions (horizontal lines) detected in reads from UDPS of HE gene (A) and F gene (B) amplicons from a non-virulent HPR0- and a virulent ISAV strain. Grey shading and the red horizontal line illustrate that only mutations present in frequencies ≥ 0.05 % are included. Color coding; light green = single screening sample, dark green = pooled screening sample, light blue = single outbreak sample, dark blue = pooled outbreak sample. The three red vertical lines in B) is the L_266_/Q_266_ codon in the F gene. Numbering of deletion events in A) correspond to numbering in Figures **2a, b**. * = deletion-insertion types observed in F reads (regions deleted are shown, see Table **S3** for details), • = outbreak delHPRs in screening samples, and♦ = full-length HPRs in outbreak samples. Numbers in bold indicate ORF_start_ and ORF_end_ positions in HPRO HE- and F genes not containing insertions. Red numbers indicate start and end of the full-length HPR, and numbers in parenthesis downstream of this region are the corresponding positions relative ORF_start_ in the outbreak delHPR. Dotted lines illustrate the full-length HPR portion not present in the delHPR.

Generally, the prevalence (i.e. # of different mutational sites compared to Sanger sequence) and frequency (i.e. # of reads containing a particular mutation differing from Sanger sequence) of mutations above 0.05 % in reads from HE amplicons (i.e. high frequency mutations) seemed higher for screening- compared to outbreak samples, as illustrated in Figure **1a** and Table **S2**. There were 22 sites in total with mutational frequency above 0.05%, 18 in screening- and 5 in outbreak samples (Table **S2**). A chi-squared test correcting for HPRO HE and delHPR HE amplicon sizes (250 and 187 nts respectively) gave p = 0.075, supporting the tendency for a higher number of mutational sites occurring in HE amplicons from screening- compared to those from outbreak. There were a total of 4209 reads containing mutations in HE amplicons with frequency above 0.05%, of which 3319 were from screening samples (Table **S2**). Comparing the total number of mutations, correcting for differences in amplicon size, showed that HE amplicons from screening samples had a higher frequency of mutational sites compared to those from outbreak (p < 0.001, chi-squared test). The majority of high frequency mutational sites were found within the HPR0 full-length HPR which constitutes 63/250 nts of the amplicon. In the single sample from screening, nine sites were in the full-length HPR, six in the rest of the amplicon (p = 0.008, chi-squared test). In the pooled sample the corresponding number was five of eight sites in total (p = 0.051, chi-squared test) (Figure **1a**, Table **S2**).

#### Half of the deletion types in HE reads are located at or in close proximity to the T_1043_-C_1044_ site

**Figure 2:**
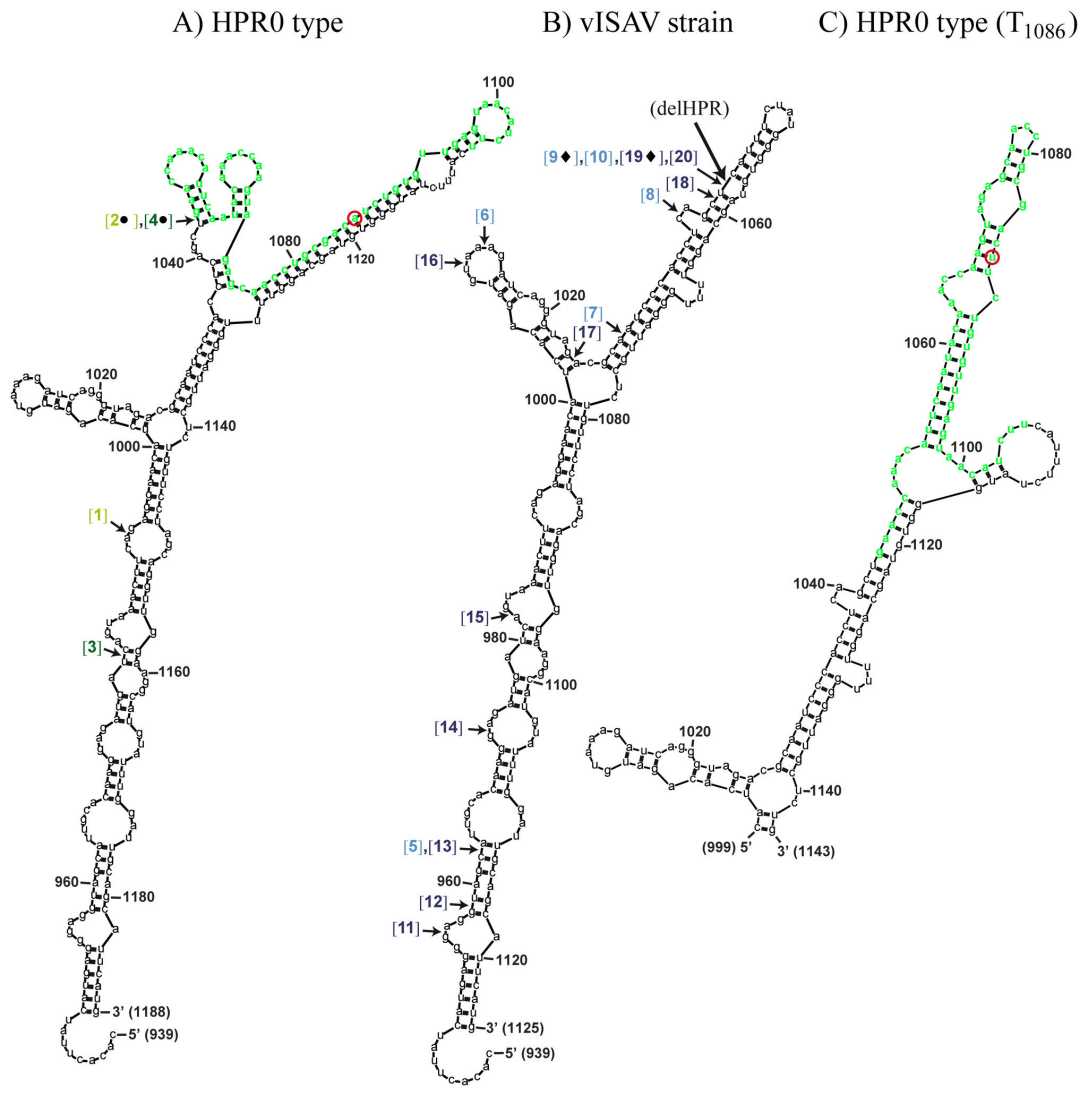
RNA secondary structure predictions of HE amplicons using mFold. The lowest free energy predicted structures of the RNAs in the sense (+) orientations are shown. A) Sanger sequence of non-virulent HPR0 strain, B) Sanger sequence of the virulent ISAV strain, and C) partial Sanger sequence from screening with a single A_1086_→T_1086_ mutation (position indicated by red circle in A) and C)). Default settings were used in the predictions, except for temperature which was set to 15°C. Sequence numbering is according to ORF_start_. The nucleotides constituting the HPR0 full-length HPR in A) and C) are shown in green color, and the site were this region is missing in the sequence from the virulent strain (between T_1043_-C_1044_) in B) is indicated by a large arrow and “delHPR”. Numbers in brackets indicate the 5’-flanking positions of deletion sites. Here, the numbering, symbols and color coding correspond to that used in Figure **1a**.

Insertions were not observed in any HE reads with the exception of the low-frequency findings of the non-deleted full-length HPR in outbreak samples (the opposite, the outbreak delHPR pattern in screening samples was also observed, see separate section). On the other hand, a number of different types of deletions were observed in HE reads, especially from outbreak samples. Although present in low frequencies (4-45 reads) they appeared mostly to have a non-random distribution. A total of fourteen different deletion types were observed for HE from outbreak samples (Figure **1a** **, 2b**, Table **S2**). Two of these are further deletions from T_1043_-C_1044_ (positions relative to start of open reading frame). This is the site in the HE gene were a putative precursor full-length HE is hypothesized to have undergone a deletion generating the outbreak delHPR pattern (i.e. the site were the Sanger sequences of full-length HPR and delHPR differ in a multiple sequence alignment). Furthermore, reads containing three other deletion types occurring immediately-, four- and fourteen nts upstream of this site, and two deletion types starting further upstream on the amplicon and ending immediately downstream of the T_1043_-C_1044_ site, were observed as well (Figures **1a**, **2b**, Table **S2**). The latter deletion type was present in both the single sample from outbreak and the pooled sample. Together, these seven deletion types (“7-dels”) all occur within fourteen nts of the T_1043_-C_1044_ site, constituting half of all deletion types observed in HE reads from outbreak samples (p < 0.001, chi-squared test). Although in some cases the differences were only a few nucleotides, none of the above deletion types produced delHPR patterns identical to that observed in outbreak strains. HE reads containing deletions starting downstream of T_1043_-C_1044_ was not observed. The remaining deletion types, both from screening- and outbreak samples, are shown in Figures **1a**, **2a**, **2b** and Table **S2**.

#### Regions flanking the delHPR T_1043_-C_1044_ site but not the full-length HPR are predicted to fold into a RNA hairpin motif

The lowest free energy predicted (+)RNA secondary structures for the Sanger sequences of the HE amplicons from screening and outbreak suggests that the full-length HPR in the former folds into a structure with less basepairing (i.e. “HPR0 RNA motif”) compared to a single hairpin motif predicted to be formed by sequences flanking the T_1043_-C_1044_ site in the latter (Figures **2a, b**). These two motifs, displayed only when the predictions were run in the sense (+) orientation, were consistently present with varying input sequence lengths up to 800 nts (the largest input length allowed in the program) (not shown). Comparing UDPS data for HE from outbreak samples with predicted RNA structure shows that the 7-dels all start or end within the predicted hairpin motif, with four starting at or close to the T_1043_-C_1044_ site in the top half of this structure (Figure **2b**). As a higher proportion of high frequency mutation sites (11) were present within the full-length HPR from screening samples, we also checked whether any of these single nucleotide variants would alter the predicted RNA structure of the Sanger sequence in this region. Tested separately, only the A_1086_→T_1086_ mutation predicted a significant structural change in (+)RNA, transforming most of the predicted HPR0 RNA motif into a single hairpin structure similar to that predicted for the delHPR (Figures **2a, c**). In the pooled samples, not a single read containing the A_1086_→T_1086_ mutation was present.

### F Gene Amplicons

#### F reads from the single screening sample contain sites with high mutation frequencies

As observed for HE reads, the prevalence and frequency of mutations in F reads above cutoff (≥ 0.05 %) were higher for screening samples compared to outbreak samples (Figure **1b**, Table **S3**). There were 25 sites in total with mutational frequency above 0.05%, 22 in screening- and 7 in outbreak samples (Table **S3**) (p = 0.013, chi-squared test) supporting also for F the tendency for a higher number of mutational sites occurring in amplicon samples from screening- compared to those from outbreak. There were a total of 7584 reads containing mutations in F amplicons with frequency above 0.05%, of which 6799 were from screening samples (Table **S3**). Similar to HE, F amplicons from screening samples has a higher frequency of mutational sites compared to those from outbreak (p < 0.001, chi-squared test). Opposed to all other samples though, the single sample from screening contained several sites displaying exceptionally high variability (≥ 1%) (Figure **1b**, Table **S3**). These sites were also present in high frequency in the pooled sample, although with a prevalence approximately 10x lower.

#### Frequencies of L_266_ codon in screening samples and Q_266_ codon in outbreak samples were not higher than at any other random site

As the codon immediately upstream of the putative encoded cleavage sites R_267_ in the F gene marks a crucial difference between HPR0s and vISAVs it was of interest to see whether the codon for this site displayed higher variability. We found that the mutation frequency in this codon, CA_797_G↔CT_797_G (Q_266_↔L_266_) was not higher than the mutation frequency at any random site in the two amplicon types (<<0,05%). Also, the A_797_ vs. T_797_ does not introduce changes in predicted (+)RNA or (-)RNA structures. In contrast, the only other site that differs between the F gene Sanger sequences from screening and outbreak, G_755_ vs. A_755_, does introduce significant changes in predicted RNA structure in the immediate region in both (+)- and (-) RNA (not shown). Hence, the contribution of this nt difference to the differing mutant spectra profiles between the two types of F amplicons, cannot be excluded.

#### Both deletions and insertions are found in F reads, the majority located in proximity to the R_267_ codon

ISAV strains with small insertions (originating from other parts of the ISAV genome) close to the putative encoded cleavage site R_267_ in the F gene have been documented [30-32]. In the present study, reads containing sequence insertions and/or deletions were observed in low frequency from both screening- and outbreak samples (4-30 reads). These are all shown in Figure **1b** and Table **S3**. Of a total of three deletion-insertion events, two are from the single screening sample where one, a 23 nt insertion, is located only 6 positions downstream of where insertions have been found in some ISAV outbreak strains. In contrast, the number of reads containing deletions of varying lengths was higher. Five of a total of eight deletion types (“5-dels”), all from outbreak samples, start at the same position (3), immediately upstream (1) or downstream (1) of were insertions have been observed in outbreak strains (at two sites separated by 11 nts). These 5-dels occur within a 12 nt region, which is a higher occurrence compared to the rest of the amplicon region (p < 0.001, chi-squared test). RNA secondary structure predictions on the Sanger sequences using mFold suggest that the 5-dels, and the 23 nt insertion type from the screening sample may, similar to that seen for the HE from outbreak (see above), occur within hairpin structures (not shown). For amplicons from screening samples, the hairpin motif was most prominent when the prediction was run on (-)RNA. Of the 5-dels from outbreak, three were located at the distal tip of a predicted hairpin, the structural feature displayed in all lowest energy predicted (+/-)RNA structures (up to the maximum allowed input sequence length of 800 nts).

### Double Infection with HPR0 and vISAV

#### DelHPR’s in screening samples and vice versa

Sequencing (Sanger) of upstream regions of the HE genes from one screening sample (screening sample 3) and one outbreak sample (outbreak sample 3) verified that the outbreak strain from Site 10 was identical to the outbreak strain from two years earlier at the same site. Together with information previously obtained from the Astafjord region [28], the sequencing showed that the HPR0 genotype at this site was not the likely precursor of the outbreak strain that emerged three weeks later (not shown). From UDPS, both the single- and pooled sample from screening, low frequency reads (21-30) containing deletion pattern identical to the Site 10 delHPR Sanger sequence, were observed (Figure **1a**, Table **S2**). The opposite was also found, reads (22,23) containing full-length HPR in outbreak samples. To confirm their presence and rule out the possibility of sample cross-contamination, two real-time PCR assays using reverse primers differentiating between the full-length HPR and the delHPR were run on all samples prior to pooling and shipping for UDPS (Table **S1**). The results, including sequencing of the real-time PCR products, verified the presence of these low frequency reads (not shown). 

## Discussion

UDPS of non-virulent ISAV HPR0- and virulent ISAV strains is presented for the first time. Gill tissues were sampled from healthy fish positive for the HPR0 strain from a screening regime, and a virulent (v) ISAV strain from a subsequent ISA disease outbreak at the same fish farming site three weeks later. HE- and F gene regions including the HPR and the codon for the putative encoded R_267_ cleavage site were amplified by PCR, pyrosequenced and detailed analysis of the sequence data was performed. The raw UDPS data revealed that roughly 10% of the reads in each sample differed from the Sanger sequences. Mutant spectra from the two ISAV strains were compared and linked to predicted viral RNA structures. 

Different mutant spectra patterns were observed for both HE- and F amplicons between the HPR0- and vISAV strains, with a higher number of high frequency mutation sites in screening than outbreak samples (see below). Moreover, HE reads from screening samples revealed a strong preference for high frequency mutations within the full-length HPR, suggesting this region to be more prone to mutations compared to the flanking regions of the amplicon. For many RNA viruses, studies have shown that nucleotide sites not forming molecular base pairs tend to show higher variability compared to those who do [54]. It should therefore not be excluded that the predicted RNA structure characteristics of the loop-containing HPR0 motif contributes to the higher frequency of mutations observed in this region. In F reads from single screening sample, the exceptionally high mutational frequency (≥ 1.0 %) observed at several positions may correlate with the fact that this sample initially contained a viral load 10-100x higher, as estimated by real-time RT-PCR (not shown), compared to the individual samples included in the pooled screening sample. On the other hand, the CT_797_G vs. CA_797_G codons (i.e. the L_266_ vs. Q_266_ virulence marker) were not found in higher frequencies in screening vs. outbreak samples, respectively, than the mutational frequency at any random position. 

Reads containing deletions were observed in both HE- and F reads while insertions were only observed in the latter. There was a higher number of different deletion types in HE reads than in F reads. For HE, half of the deletion types from outbreak samples (7 of 14) start or end within a fourteen nt region, where two are further deletions from T_1043_-C_1044_, the site where the original deletion from a precursor HPR0 genotype can be hypothesized to have occurred. Similarly, of a total of ten different insertion-deletion- or deletion types found in F reads, three were found to start at the same position were most F gene insertions have been found in outbreak strains [30-32]. It cannot be excluded that several of these events have been artificially generated during the PCR amplification step or during UDPS [55,56]. However, studies have indicated that the *in vitro* recombination rate in UDPS is low [56]. For HE, this suggests a tendency towards further deletions from T_1043_-C_1044_ following a primary deletion event, possibly linked to increased viral fitness associated with larger deletions. In fact, closely related vISAVs isolated in the course of the same outbreak at the same fish farming site have been found to differ only by the size of their deletions in the HPR, where one isolate is likely to have originated from a further deletion of the other [57]. 

The results obtained from UDPS suggest a link between mutant spectra profiles and the predicted hairpin motifs observed with Sanger sequences of HE (+)RNA and F (+/-)RNAs from outbreak, and F (-)RNA from screening. Such a RNA structure may facilitate mutational events such as insertions (F) and deletions (HE) through mechanisms such as RNA polymerase jumping- and/or template switching during replication. For influenza A viruses, jumping of the RNA polymerase has been suggested as a mechanism for generating deletions and insertions in hemagglutinin and neuraminidase genes [58]. The involvement of RNA structure in the generation of the polybasic cleavage site in highly pathogenic avian influenza A strains, by the polymerase slipping during strand synthesis upon arrival at a region of higher stability, has also been suggested [59]. For F genes, a sequence-based non-homologous replicase-driven template switch, as observed in positive-stranded RNA viruses, has been proposed as the most likely mechanism behind the sequence insertions observed in some vISAVs [30,60]. The inserted sequences in F genes were all found to originate from other parts of the ISAV genome, and not the host, we believe most likely because these sequences are in close proximity to one another, associated with replicase complex, during ISAV replication in the nucleolus [61].

From the present UDPS data, only one deletion-insertion event (F reads, pooled outbreak sample) and one deletion event (HPRO HE reads, single screening sample) seem to have been generated through the involvement of extensive sequence homology (not shown), although whether these were generated artificially or are true quasispecies, is not known. In general though, recombination is known to be rare in negative strand- compared to positive-stranded RNA viruses like *Picornaviridae* and *Coronaviridae* [60,62]. Our results from UDPS data- and RNA secondary structure prediction analysis suggests that RNA structural properties may be involved in generating the F gene insertions, supported also by the fact that non-homologous recombination is very rare in orthomyxoviruses. For ISAV HE, based on the results from both UDPS and RNA structure predictions, a plausable mechanism for creating the deletions in HE is likely to involve the replicase complex bypassing a sequence stretch on the same template or jumping to a homologous template reinitiating strand synthesis further downstream. This event is hypothesized to be driven through alterations in RNA structure (i.e. hairpin structure) following point mutations in the full-length HPR. The distributions of deletions and the total absence of insertions in HE reads together with current epidemiological information on vISAVs supports this hypothesis. Compared to previous publications on this issue, the present results strongly suggest that the recombination mechanisms creating the deletions do not involve insertions nor recombination between HPR regions between different virulent strains [14]. 

The high frequency of mutations within the full-length HPR of screening samples prompted investigation of whether any of these mutations (11 in all) tested individually would change the predicted (+/-)RNA structure of the Sanger HPR0 motif. Results from several studies suggest a direct link between the structure of viral RNA and the fidelity of the RNA polymerase [54]. Only one mutation, A_1086_ to T_1086_, found in reads from the single screening sample resulted in a change in the predicted structure, transforming most of the predicted HPR0 motif into hairpin-like structure. Hence, single mutations in the full-length HPR may have the potential to transform this RNA motif into one resembling the regions flanking the delHPR site, potentially facilitating deletions in this region. In fact, for avian influenza A viruses the generation of the polybasic cleavage site characteristic of high pathogenic strains has been suggested also to be mediated by a mechanism in which single nucleotide changes alter RNA secondary structure facilitating the introduction of small sequence insertions in this region [63]. Here, aside from the fact that the HPR from the single screening sample contained roughly twice the amount of high frequency mutation sites compared to the pooled sample, it is noteworthy that not a single read from the pooled sample contained the T_1086_ mutation. This might reflect a link between the severity of infection by a HPR0 strain, possibly influenced by a high-stress fish farming environment, and the composition of mutant spectra. Studies have shown that differences in HPR0 viral loads between individuals is linked to the point in time of infection and that the virus may be present, although transiently, in the population for some time, with no clinical signs of ISA [27]. The effect may thus be a broadening of the mutant spectra in HPR0 quasispecies in spite of low replication rate for these non-virulent strains. In contrast, upon mutation leading to vISAVs, one sequence that harbours the mutation will outcompete the other sequences and spread as a single strain displaying narrower mutant spectra, at least in the early stages of the disease outbreak. In the present study, sampling of the vISAV strain was performed in the early phases of the disease outbreak and displayed many magnitudes higher viral loads compared to the samples from screening. 

We hypothesize that the mutant spectra displayed by HPR0 infections in a fish farming environment is directly linked to the potential for deletions in the full-length HPR, and that RNA structure plays a central role in this transition. Also, the observed differences in mutant spectra by the two ISAV strains (like the higher number of high frequency mutation sites in screening vs. outbreak samples) may not only be linked to differences in infection period but also to replication rates, as well as to possible variations in the viral RdRps that may affect both replication rate and replication fidelity, thus changing quasispecies populations and hence viral fitness [42,64-68]. Hence, the potential involvement of sequence differences in internal genes on the differential mutational frequencies observed between the HPR0- and vISAV strains cannot be excluded. Also, the putative gill tropism displayed by HPR0s in farmed and wild Atlantic salmon compared to the systemic nature of vISAV infections, possibly involving different cell types in the gills [69] and different parts of the immune system, may also be a contributing factor to the observed differences in the mutant spectra. As ISA has not been observed in wild Atlantic salmon, a future comparative study between mutant spectra from HPR0-positive wild salmon with that obtained in the present study could provide valuable insight into the mechanisms of ISAV transition into virulence. It should also be considered that Atlantic salmon may not be the natural host for HPR0s and the transition to vISAV could be viewed as an adaptation of the virus in this particular host.

This study represents the first documentation of a double infection involving non-virulent HPR0s and vISAVs. The low number of delHPR reads in screening samples corresponds well with the number of reads containing L_266_ codons, as do the low number of full-length HPRs vs. Q_266_ codons from outbreak samples. Although statistically unlikely, it cannot be excluded that one or several of the deletions observed in HE- and F amplicons are linked to the low frequency presence of the other ISAV variant. For single site mutations though, the threshold level set eliminates this potential misinterpretation. From Sanger sequencing of larger portions of the HE genes it was deemed unlikely that the HPR0 strain from the screening samples was the direct precursor of the vISAV strain in this particular fish farm. Hence, many individuals showing no signs of ISA disease were already infected with a virulent strain at the time of screening, suggesting that infection with a HPR0 strain may not protect the fish from ISA disease. Also, vaccination against ISA has shown little or no protection against infection with HPR0s [27]. The opposite finding, i.e. the presence of full-length HPRs in outbreak samples, could either be due to the fact that sampling was performed in the early stages of the disease outbreak, or that an earlier infection with a HPR0 virus persists for some time at basal levels in ISA-diseased individuals caused by a virulent strain. Such double infections may actually play an important role in ISAV evolution.

UDPS technology has shown itself to be a valuable tool in detecting minority within-host mutant variants of viral genomes not detected by traditional means, such as low-level persistence of drug resistant mutations in individuals following prophylactic treatment, or double infections [44,70-73]. The latter is also the case here, where the low-frequency presence of delHPRs in screening samples were not detected through the standard diagnostic procedures involving RT-PCR and Sanger sequencing, thus demonstrating the future potential UDPS technology may have in ISAV screening regimes and diagnostics.

In conclusion, we have performed UDPS of HE- and F variable gene regions from samples containing a non-virulent HPR0 strain and a virulent ISAV strain. Detailed analysis and comparisons of mutant spectra from both individual and pooled samples revealed marked differences between the two virus strains. We propose a new hypothesis were the introduction of selected mutations in the full-length HPR can alter the RNA structure in such a way that facilitates deletion events in this gene region, making both quasispecies composition and RNA structure important factors involved in the two known molecular events leading to ISAV virulence. The results further strengthen the hypothesis that deletions, and not recombination, form the variability of delHPRs in virulent ISAVs and support observations from Norway and the Faroe Islands that this transition to virulence is an infrequent event.

## Supporting Information

Table S1 **PCR primers and probes.**
(DOCX)

Table S2 **Type, number and prevalence of mutations present in ≥ 0.05% of reads and deletions as detected by UDPS of HE amplicons.**
(DOCX)

Table S3 **Type, number and prevalence of mutations present in ≥ 0.05% of reads, deletions and insertions as detected by UDPS of F amplicons.**
(DOCX)
