# Supplementary material for: Ultra-Deep Pyrosequencing of Partial Surface Protein Genes from Infectious Salmon Anaemia Virus (ISAV) Suggest Novel Mechanisms Involved in Transition to Virulence
Source: PLoS One. 2013 Nov 26;8(11):e81571. doi: 10.1371/journal.pone.0081571 (PMC3841194; doi:10.1371/journal.pone.0081571)
Supplement: Table S1 — PCR primers and probes. (DOCX) [file pone.0081571.s001.docx]

**Table S1:** PCR primers and probes.

| Assay type | Primer/probe name | Primer/probe sequence (5’-3’) |
| --- | --- | --- |
| Pyrosequencing^a^ | s6 HPR_Fpyro | CGTATCGCCTCCCTCGCGCCA*TCAG*ACGAGTG CGT**G_913_ACCAGACAAGCTTAGGTAACACAGA** |
|  | s6 HPR_Rpyro | CTATGCGCCTTGCCAGCCCGC*TCAG*acgctcga ca**G_1217_ATGGTGGAATTCTACCTCTAGACTTGTA** |
|  | s5 cleav_Fpyro | CGTATCGCCTCCCTCGCGCCA*TCAG*AGACG CACTC**G_683_AGTAGTGCCGTTCCATTCTGTAC** |
|  | s5 cleav_Rpyro | CTATGCGCCTTGCCAGCCCGC*TCAG*AGC ACTGTAG**T_920_CTTCTGCGGATGCTGCACC** |
| Real-time PCR 1^b^ | s6HPR_F | GACCAGACAAGCTTAGGTAACACAGA |
|  | s6delHPR_R | CATAGAAATGAGCTGAGGTGGGAT |
|  | s6delHPR_MGB | (6-FAM)ACCTCCCTCATGATAAGT(MGBNFQ) |
| Real-time PCR 2^c^ | s6HPR0_F | CTCAGCTGAACCAAACATTCAATACA |
|  | s6HPR0_R | GAAATGAAGATGTTACTCAACACAGATGT |
|  | s6HPR0_MGB | (6-FAM)ACCAAGTAGAGCAACCTG(MGBNFQ) |

^a^Template specific sequences in segment 6 (HE gene) and segment 5 (F gene) primers are in bold. The remaining are sequences necessary for pyrosequencing in both directions and multiplexing; adaptor sequences (normal font), library keys (italics) and multiplex identifiers (MIDs) (underlined). Lower case letters indicate positions in HE HPR0- and F gene ORFs, respectively.

^b^Real-time PCR for detection of low-frequency delHPR reads in screening samples.

^c^Real-time PCR for detection of low-frequency full-length HPR reads in outbreak samples.
