# Supplementary material for: Ultra-Deep Pyrosequencing of Partial Surface Protein Genes from Infectious Salmon Anaemia Virus (ISAV) Suggest Novel Mechanisms Involved in Transition to Virulence
Source: PLoS One. 2013 Nov 26;8(11):e81571. doi: 10.1371/journal.pone.0081571 (PMC3841194; doi:10.1371/journal.pone.0081571)
Supplement: Table S2 — Type, number and prevalence of mutations present in ≥ 0.05% of reads and deletions as detected by UDPS of HE amplicons. (DOCX) [file pone.0081571.s002.docx]

**Table S2:** Type, number and prevalence of mutations present in ≥ 0.05% of reads and deletions as detected by UDPS of HE amplicons.

| HE amplicon | Shared mutations in forward and reverse | Deletions in reads^d^ |
| --- | --- | --- |
| sample(s) | reads with prevalence ≥ 0.05% |  |
|  | (direction of read: # of reads/% frequency)^c^ |  |
| SCN1^a^ | C969T (F:141/0.28, R:155/0.22) | p993→p1094 (1) |
|  | A1018G (F:52/ 0.10, R 63/0.09) | p1044→ p1106 (2•)^e^ |
|  | **A1057G (F:25,/0.05, R 38/0.05)** |  |
|  | **A1064C (F:60/0.12, R:69/0.10)** |  |
|  | **A1077G (F:134/0.27, R:151/0.22)** |  |
|  | **T1080C (F:41/0.08, R:42/0.06)** |  |
|  | **C1082T (F:27/0.05, R:45/0.06)** |  |
|  | **C1085A (F:133/0.26, R:151/0.22)** |  |
|  | **A1086T (F:136/0.27, R:152/0.22)** |  |
|  | **T1089C (F:50/0.10, R:48/0.07)** |  |
|  | **T1098C (F:135/0.27, R:149/0.21)** |  |
|  | A1125G (F:31/0.06, R:55/0.08) |  |
|  | T1131G (F:63/0.12, R:81/0.12) |  |
|  | G1161A (F:112/0.22, R:164/0.24) |  |
|  | C1178T (F:25/0.05, R:40/0.06) |  |
| SCN pool | C1004T (F:14/0.05, R: 46/0.07) | p981→p1102 (3) |
| (SCN2, SCN3, SCN4) | A1018G (F: 26/0.10, R: 59/0.09) | p1044→ p1106 (4•)^f^ |
|  | **A1067G (F:61/0.24, R:164/0.24)** |  |
|  | **C1078A (F:28/0.11, R:(65/0.09)** |  |
|  | **T1080C (F:27/0.11, R:38/0.06)** |  |
|  | **C1082T (F:18/0.07, R:35/0.05)** |  |
|  | **T1089C (F:27/0.11, R:46/0.07)** |  |
|  | T1131G (F:26/0.10, R:71/0.10) |  |
| OBK1^b^ | A1018G (F:37/0.07, R:74/0.08) | p963→p1044 (5) |
|  | G1027A (F:40/0.08, R:55/0.06) | p1013→p1044 (6) |
|  | T1050C (F:30/0.06, R:55/0.06) | p1030→p1062 (7) |
|  | A1062G (F:42/0.08, R:54/0.06) | p1040→p1066 (8) |
|  | T1068G (F:54/0,10, R:95/0,11) | p1044→ p1106 (9♦)^g^ |
|  |  | p1044→p1058 (10) |
| OBK pool | A1018G (F:33/0.08, R:50/0.10) | p956→p1088 (11) |
| (OBK2, OBK3, OBK4) | G1027A (F:24/0.06, R:40/0.08) | p956→p1061 (12) |
|  | T1050C (F:34/0.08, R:30/0.06) | p963→p1044 (13) |
|  | A1062G (F:21/0.05, R:28/0.05) | p974→p1022 (14) |
|  | T1068G (F:31/0.08, R:63/0.12) | p983→p1020 (15) |
|  |  | p1011→p1019 (16) |
|  |  | p1025→p1034 (17) |
|  |  | p1043→p1061 (18) |
|  |  | p1044→ p1106 (19♦)^h^ |
|  |  | p1044→p1052 (20) |

^a^SCN = screening sample. Mutations within the HPR0 full-length HPR are shown in bold.

^b^OBK = outbreak sample.

^c^Numbering according to start of open reading frame (ORF_start_).

^d^Reads containing insertions were not observed.

^e,f^Site 10 outbreak delHPR.

^g,h^Full-length HPR (numbering according to ORF_start_ for HPR0s).

Numbers and symbols in parenthesis in the right column (1-20) correspond to that used in **Figures 1a, 2a and 2b**.
