# Supplementary material for: Ultra-Deep Pyrosequencing of Partial Surface Protein Genes from Infectious Salmon Anaemia Virus (ISAV) Suggest Novel Mechanisms Involved in Transition to Virulence
Source: PLoS One. 2013 Nov 26;8(11):e81571. doi: 10.1371/journal.pone.0081571 (PMC3841194; doi:10.1371/journal.pone.0081571)
Supplement: Table S3 — Type, number and prevalence of mutations present in ≥ 0.05% of reads, deletions and insertions as detected by UDPS of F amplicons. (DOCX) [file pone.0081571.s003.docx]

**Table S3:** Type, number and prevalence of mutations present in ≥ 0.05% of reads, deletions and insertions as detected by UDPS of F amplicons.

| F amplicon | Shared mutations in forward and reverse | Deletions/insertions |
| --- | --- | --- |
| sample(s) | reads with prevalence ≥ 0.05%. | in reads |
|  | (direction of read: # of reads/% frequency)^c^ |  |
| SCN1^a^ | G754A (F:455/1.00, R:534/1.10) | p810→p821 (*)^d^ |
|  | G755A (F:478/1.10, R:536/1.10) | p833→p851 (*)^e^ |
|  | C765A (F:46/0.10, R:53/0.11) |  |
|  | A768G (F:272/0.61, R:321/0.65) |  |
|  | A788G (F:450/1.01, R:534/1.10) |  |
|  | A810G (F:29/0.07, R:31/0.06) |  |
|  | G849T (F:166/0.37, R:193/0.39) |  |
|  | T867C (F:178/0.40, R:202/0.41) |  |
|  | A888G (F:287/0.64, R:346/0.70) |  |
|  | G889A (F:222/0.50, R:315/0.64) |  |
|  | A893G (F:35/0.08, R:46/0.09) |  |
| SCN pool | C715A (F:22/0.07, R:19/0.05) |  |
| (SCN2, SCN3, SCN4) | C715T (F:37/0.12, R:51/0.14) |  |
|  | G724A (F:27/0.09, R:26/0.07) |  |
|  | T726C (F:27/0.09, R:31/0.09) |  |
|  | C752T (F:42/0.13, R:38/0.11) |  |
|  | G754A (F:30/0.10, R:34/0.09) |  |
|  | G755A (F:29/0.09, R:52/0.15) |  |
|  | G760A (F:32/0.10, R:18/0.05) |  |
|  | A788G (F:29/0.09, R:42/0.11) | - |
|  | T791C (F:18/0.06 R:24/0.07) |  |
|  | A810G (F:31/0.10, R:22/0.06) |  |
|  | T832C (F:19/0.06, R:27/0.08) |  |
|  | G833A (F:25/0.08, R:19/0.05) |  |
|  | G849T (F:16/0.05, R:29/0.08) |  |
|  | C852T (F:48/0.15, R:51/0.14) |  |
|  | T867C (F:24/0.08, R:37/0.10) |  |
|  | A869G (F:16/0.05, R:21/0.06) |  |
|  | A893G (F:28/0.09, R:29/0.08) |  |
| OBK1^b^ | T780C (F:16/0.05, R:24/0.05) | p784→p823 |
|  | A793G (F:18/0.06, R:35/0.07) | p804→p842 |
|  | T797C (F:37/0.12, R:52/0.11) |  |
|  | A810G (F:21/0.07, R:33/0.07) |  |
|  | T832C (F:27/0.09, R:29/0.06) |  |
| OBK pool | G724A (F:27/0.06, R:31/0.07) | p742→p754 |
| (OBK2, OBK3, OBK4) | T726C (F:31/0.07, R:29/0.07) | p743→p765 (*)^f^ |
|  | A793G (F:44/0.10, R:44/0.10) | p795→p827 |
|  | T797C (F:56/0.12, R:56/0.13) | p796→p805 |
|  | A810G (F:29/0.06, R:32/0.07) | p804→p861 |
|  | T832C (F:23/0.05, R:30/0.07) | p804→p842 |
|  | A893G (F:29/0.06, R:32/0.07) | p805→p826 |

^a^SCN = screening sample.

^b^OBK = outbreak sample.

^c^Numbering according to start of open reading frame (ORF_start_).

^d^Deletion replaced by 23 nt insertion.

^e^Deletion replaced by 25 nt insertion.

^f^Deletion replaced by 20 nt insertion.

Asterisks in parenthesis in the right column correspond to that used in **Figure 1b**.
